# Bewegungsförderung mittels digitaler Technologien: Körperliche Alltagsaktivität und psychische Gesundheit

**DOI:** 10.1007/s00103-026-04320-9

**Published:** 2026-10-19

**Authors:** Carlotta Kunst, Dominic Reichert, Johanna Rehder, Bernd Lenz, Marco Giurgiu, Markus Reichert

**Affiliations:** 1 Klinik für Psychiatrie und Psychotherapie, Zentralinstitut für Seelische Gesundheit, Medizinische Fakultät Mannheim, Universität Heidelberg, Mannheim, Deutschland; 2 AG Sportpsychologie und Sportsoziologie, FB Sport- und Bewegungswissenschaft, Natur- und Lebenswissenschaftliche Fakultät, Universität Salzburg, Schlossallee 49, 5400 Hallein, Österreich; 3 Junior Researcher Group eHealth and Sports Analytics, Fakultät für Sportwissenschaft, Ruhr-Universität Bochum, Bochum, Deutschland; 4 Klinik für Abhängiges Verhalten und Suchtmedizin, Zentralinstitut für Seelische Gesundheit, Medizinische Fakultät Mannheim, Universität Heidelberg, Mannheim, Deutschland; 5 Standort Mannheim-Heidelberg-Ulm, Deutsches Zentrum für Psychische Gesundheit, Mannheim, Deutschland; 6 Institut für Bewegungstherapie und bewegungsorientierte Prävention und Rehabilitation, Deutsche Sporthochschule Köln, Köln, Deutschland

**Keywords:** Bewegungsförderung, Ambulantes Assessment, Wearables, Just-in-Time Adaptive Interventions, Digitale Gesundheitsanwendungen, Physical activity promotion, Ambulatory assessment, Wearable devices, Just-in-time adaptive interventions, Digital therapeutics

## Abstract

Körperliche Aktivität ist essenziell für die körperliche und psychische Gesundheit. Dennoch werden Bewegungsintentionen im Alltag häufig nicht umgesetzt. Zu dieser Intentions-Verhaltens-Lücke können Gewohnheiten, situative Einflüsse und momentanes Erleben beitragen. Digitale Technologien ermöglichen es, diese Faktoren und das Bewegungsverhalten alltagsnah zu erfassen und darauf abgestimmte Interventionen anzubieten.

Der narrative Übersichtsbeitrag erläutert zunächst anhand verschiedener Modelle den Einfluss reflektierter und automatischer Prozesse auf das Bewegungsverhalten. Anschließend werden das Ambulante Assessment zur wiederholten Erfassung von Bewegung und psychischem Erleben im Alltag und Wearables als technologische Grundlage der passiven Datenerfassung vorgestellt. Zudem werden Just-in-Time Adaptive Interventions (JITAIs) beschrieben, die zu geeigneten Zeitpunkten Bewegungsimpulse geben können. An 2 Beispielprojekten aus der Sucht- und Depressionsforschung wird veranschaulicht, wie solche Impulse körperliche Aktivität anregen und klinisch relevante Merkmale wie Craving (Suchtdruck), subjektive Energie oder Antrieb beeinflussen könnten. Zudem werden Digitale Gesundheitsanwendungen (DiGA) als möglicher Weg in die Regelversorgung diskutiert.

Chancen digitaler Bewegungsförderung liegen in der zeitnahen und individuellen Unterstützung. Limitationen betreffen Messgenauigkeit und Datenqualität, die langfristige Nutzung sowie den bislang begrenzten Stellenwert eigenständiger Bewegungsförderung in digitalen Anwendungen für psychische Erkrankungen. Potenzial bieten Blended-Care-Ansätze, bei denen digitale Angebote die persönliche Behandlung alltagsnah ergänzen.

## Abstract (en)

Physical activity is essential for physical and mental health. Yet intentions to be active often do not translate into action in everyday life. Habits, situational influences and momentary experiences may contribute to this gap between intention and behaviour. Digital technologies can capture these factors and physical activity in daily life and support tailored interventions.

This narrative review first outlines models of reflective and automatic influences on physical activity. It then introduces ambulatory assessment for repeatedly measuring physical activity and psychological experiences in daily life and wearables for passive data collection. Just-in-time adaptive interventions (JITAIs), which can provide activity prompts at suitable moments, are also described. Two research projects on addiction and depression illustrate how these prompts might encourage physical activity and influence clinically relevant features such as craving, subjective energy or drive. Digital health applications (DiGA) are discussed as a potential route into routine care.

Digital physical activity promotion offers timely and individualized support. Limitations include measurement accuracy, data quality, sustained use and the limited role of dedicated physical activity promotion in digital applications for mental disorders. Blended care approaches, in which digital tools complement face-to-face treatment in everyday life, offer potential.

## Einleitung: Bewegung und psychische Gesundheit im digitalen Zeitalter

Eine salutogenetische, also ressourcenorientierte Perspektive richtet den Blick nicht allein auf die Vermeidung von Krankheit, sondern auf Ressourcen, die Gesundheit erhalten und stärken. Körperliche Aktivität kann als eine solche Ressource verstanden werden [1]. Körperliche Inaktivität ist etwa mit höherem Risiko für Typ-2-Diabetes, koronare Herzkrankheit und Schlaganfall verbunden [2], während höhere körperliche Aktivität zum Beispiel mit einem geringeren Risiko für die Entwicklung depressiver Störungen assoziiert ist [3].

Auch kleine, realistisch erreichbare Steigerungen könnten populationsbezogen relevant sein: Einer Metaanalyse zufolge wären bei 5 zusätzlichen Minuten moderater bis intensiver körperlicher Aktivität pro Tag, je nach zugrunde gelegter Population und Strategie, etwa 6–10 % der Todesfälle potenziell vermeidbar [4]. Würden Erwachsene weltweit die Empfehlungen der Weltgesundheitsorganisation (WHO) von mindestens 150 min moderater Aktivität pro Woche erfüllen, könnte das globale Bruttoinlandsprodukt im Jahr 2050 schätzungsweise um 314–446 Mrd. US-Dollar höher liegen [5]. Eine Metaanalyse alltagsnaher Daten zeigt zudem, dass körperliche Aktivität innerhalb einer Person sowohl mit vorausgehendem als auch mit nachfolgendem momentanen Wohlbefinden zusammenhängt; am stärksten war die Beziehung zum Gefühl, energiegeladen und wach zu sein [6].

Das Wissen um die gesundheitsförderliche Wirkung von Bewegung führt nicht automatisch dazu, dass Menschen dauerhaft aktiver werden. Ob eine Bewegungsintention im Alltag tatsächlich umgesetzt wird, kann von wechselnden affektiven, automatischen und situativen Einflüssen abhängen [7, 8]. Die Diskrepanz zwischen Verhaltensintention und tatsächlichem Verhalten wird als Intentions-Verhaltens-Lücke beschrieben [8, 9]. Für die Bewegungsförderung ist es deshalb wichtig, affektive, automatische und situative Einflüsse dort zu erfassen, wo Bewegungsverhalten entsteht: im Lebensalltag.

Der hier vorliegende Beitrag fokussiert vor allem nichtsportliche körperliche Alltagsaktivität. Gemeint sind spontane oder gewohnheitsmäßige Bewegungen, die nicht als strukturiertes Training geplant sind, sondern beispielsweise beim Gehen, bei aktiver Mobilität mit dem Fahrrad, bei der Gartenarbeit oder im Haushalt entstehen [10]. Da sie nicht an feste Trainingszeiten oder bestimmte Orte gebunden sein müssen, bieten solche Aktivitäten einen niedrigschwelligen Ansatzpunkt für Bewegungsförderung.

Digitale Technologien ermöglichen es, Bewegungsverhalten und aktuelles Erleben in Alltagssituationen wiederholt und zeitnah zu erheben: Kurze Smartphone-Befragungen erfassen psychologische Variablen wie Stimmung, am Körper getragene Sensoren das Bewegungsverhalten [7]. Zusammen können diese Informationen als Grundlage dienen, Bewegungsinterventionen in bestimmten Situationen im Alltag anzubieten [7, 11, 12].

Bei psychischen Erkrankungen kann dieser Ansatz besonders relevant sein. Zum Beispiel bietet bei depressiven Störungen der Zusammenhang zwischen körperlicher Alltagsaktivität und subjektiver Energie sowie subjektivem Antrieb aktuellen Studien zufolge möglicherweise eine Chance für innovative digitale Bewegungsinterventionen. Bei Alkoholgebrauchsstörungen könnten beispielsweise Situationen mit erhöhtem Suchtdruck (Craving) oder Einsamkeit Ansatzpunkte für alltagsnahe Bewegungskurzinterventionen bieten [6, 13, 14]. Aktuelle Forschungsprojekte untersuchen, ob digitale Bewegungsimpulse körperliche Aktivität anregen und ob diese wiederum Energie, Craving oder andere klinisch relevante Merkmale verändern kann [12–14].

Ziel dieser narrativen Übersicht ist es, anhand ausgewählter Literatur darzustellen, wie digitale Technologien zur alltagsnahen und situationsangepassten Bewegungsförderung bei psychischen Erkrankungen beitragen können. Zunächst werden die theoretischen und methodischen Grundlagen erläutert. Anschließend wird anhand von 2 Beispielprojekten (C05 und MASE [Motor Activity - Subjective Energy]) die 2‑stufige Prüfung digitaler Bewegungsimpulse bei Alkoholgebrauchsstörungen und depressiven Störungen veranschaulicht. Im abschließenden Teil werden Digitale Gesundheitsanwendungen (DiGA) als möglicher Transferweg in die Versorgung eingeordnet, bevor Chancen, Limitationen und Perspektiven diskutiert werden.

## Theoretischer Hintergrund: Dual-Process-Modelle der Verhaltensänderung

Traditionelle sozialkognitive Modelle der Verhaltensänderung erklären, wie Intentionen durch rationale Entscheidungen entstehen; aktuell stehen sie jedoch in der Kritik, nur begrenzt zu erfassen, warum die Umsetzung der Intentionen im Alltag häufig scheitert [8]. Dual-Process-Modelle verstehen Verhalten dagegen als Zusammenspiel reflektiver, also bewusst abgewogener, und automatischer Prozesse. Reflektive Prozesse wie das Bilden einer Bewegungsintention oder bewusste Selbstregulation beanspruchen Aufmerksamkeit; automatische Prozesse beruhen auf erlernten Verknüpfungen und Gewohnheiten und laufen mit wenig bewusster Kontrolle ab [15].

**Abb. 1:**
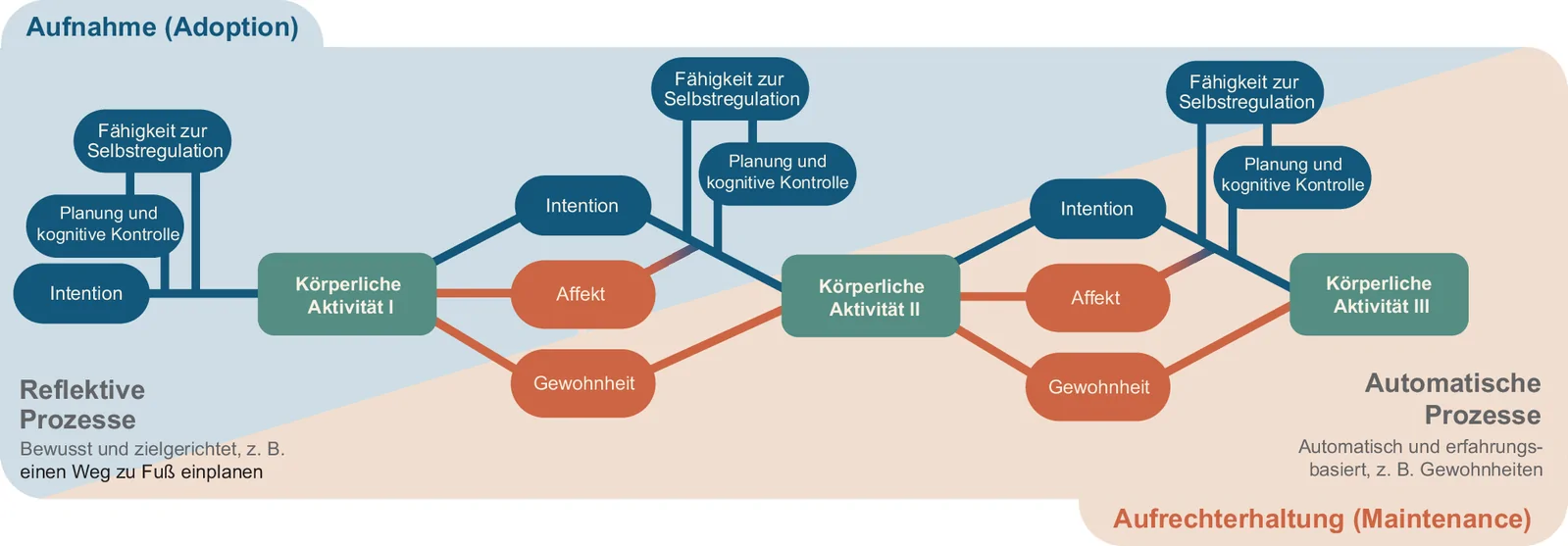
Physical Activity Adoption and Maintenance Model (PAAM). Vereinfachte Darstellung des Zusammenspiels reflektiver und automatischer Prozesse bei der Aufnahme und Aufrechterhaltung körperlicher Aktivität. Die Darstellung hebt hervor, dass reflektive Prozesse insbesondere für die Aufnahme körperlicher Aktivität bedeutsam sind, während Affekt und Gewohnheiten im Verlauf der Aufrechterhaltung an Bedeutung gewinnen. Affekt bezeichnet hier die unmittelbare emotionale Bewertung und das Erleben von Bewegung. Quelle: eigene Darstellung auf Grundlage des „Physical Activity Adoption and Maintenance Model“ (PAAM) von Strobach et al. [16]

Das Physical Activity Adoption and Maintenance Model (PAAM) überträgt diesen Grundgedanken auf körperliche Aktivität. Dem Modell zufolge sind für die Aufnahme (Adoption) vor allem reflektive Prozesse wie Bewegungsintention bedeutsam, während bei der Aufrechterhaltung (Maintenance) automatische Prozesse wie Gewohnheiten und affektive Reaktionen, also das unmittelbare Erleben und Bewerten von Bewegung, an Bedeutung gewinnen können. Für die Bewegungsförderung legt dies nahe, neben Intentionen auch Alltagssituationen und positive Bewegungserfahrungen einzubeziehen, die Ausführung und Wiederholung unterstützen [16]. Das PAAM ist in Abb. 1 schematisch dargestellt.

Das „24-hour cognitive-affective physical behavior model“ (24-Stunden-Modell des kognitiv-affektiven Bewegungsverhaltens) nimmt den gesamten Tagesverlauf in den Blick. Es verknüpft körperliche Aktivität, sitzendes Verhalten und Schlaf mit gedanklichen und gefühlsbezogenen Einflussfaktoren sowie gesundheitlichen Folgen und berücksichtigt wechselseitige Beziehungen über unterschiedliche Zeiträume [17].

Gemeinsam verdeutlichen die Modelle, warum Bewegungsintentionen nicht immer in entsprechendes Verhalten umgesetzt werden: Während Intentionen relativ stabil sein können, wird das tatsächliche Bewegungsverhalten zusätzlich durch wechselnde affektive Reaktionen, Gewohnheiten und situative Bedingungen beeinflusst [16]. Für die digitale Bewegungsförderung ist diese zeitliche und kontextuelle Dynamik zentral. Digitale Erhebungsverfahren können Veränderungen in Bewegung, Erleben und Kontext wiederholt im Alltag erfassen und damit die Grundlage für situationsangepasste Bewegungsimpulse schaffen [7].

## Methodische Innovationen in der Bewegungsforschung

### Ambulantes Assessment und digitale Intervention

Affektive, automatische und situative Einflüsse lassen sich mit Laborstudien und retrospektiven Fragebögen nur begrenzt erfassen. Künstliche Untersuchungssituationen können Erleben, Verhalten und physiologische Messwerte verzerren [18]. Beim Weißkittel-Effekt ist etwa der in einer medizinischen Umgebung gemessene Blutdruck höher als außerhalb dieser Situation [19]. Rückblickende Angaben sind zudem anfällig für Erinnerungsverzerrungen: Jüngere, besonders auffällige oder zur aktuellen Stimmung passende Ereignisse werden zum Beispiel leichter erinnert [20, 21].

Ambulantes Assessment bezeichnet die wiederholte Erfassung von Verhalten, Erleben, physiologischen Prozessen und Kontext im natürlichen Lebensumfeld [22]. Diese Erhebung verringert Laborverzerrungseffekte und erhöht die ökologische Validität, also die Gültigkeit der Studienergebnisse für den Alltag. Zeitnahe elektronische Selbstberichte über das Smartphone werden innerhalb des Ambulanten Assessments oft als Ecological Momentary Assessment (EMA) bezeichnet. Durch ihre Nähe zum Erleben verringern sie Erinnerungsfehler [18, 20]. In Verbindung mit Bewegungsdaten können sie Untersuchungen dazu ermöglichen, wann, unter welchen Bedingungen und mit welchem Erleben Bewegung stattfindet.

Wiederholte Messungen erzeugen intensive Längsschnittdaten mit vielen Messzeitpunkten pro Person und ermöglichen es, Veränderungen innerhalb einer Person zu untersuchen. Diese sogenannte Within-Person-Perspektive ist für die Alltagsforschung zentral, weil Zusammenhänge innerhalb einer Person von Zusammenhängen zwischen verschiedenen Personen abweichen können [7, 23].

Ein Beispiel verdeutlicht diesen Unterschied: Körperlich aktivere Menschen weisen im Durchschnitt häufig einen niedrigeren Blutdruck auf (Between-Subject-Perspektive). Bei derselben Person steigt der Blutdruck unmittelbar nach körperlicher Aktivität, etwa beim Treppensteigen, an (Within-Subject-Perspektive). Ergebnisse aus Gruppenvergleichen können deshalb nicht auf Prozesse innerhalb einer Person übertragen werden [24].

Ambulantes Assessment ist meist multimodal und verbindet aktive Selbstberichte mit passiv erfassten Sensordaten. Elektronische Tagebücher können affektive Reaktionen, Energie, Belastung und sozialen Kontext abbilden, während am Körper getragene Sensoren zeitgleich Bewegung und physiologische Veränderungen erfassen [25, 26]. Die Verknüpfung kann Untersuchungen dazu ermöglichen, ob, wann und unter welchen Bedingungen Bewegung stattfindet und wie sie erlebt wird [7].

Beobachtende Studien zum Ambulanten Assessment können zeitliche Abfolgen sichtbar machen, erlauben aber keine belastbaren Aussagen über Ursache und Wirkung [13, 27].

Das Within-Person-Encouragement Design (WPED) ergänzt das Ambulante Assessment um eine experimentelle Komponente: Dieselbe Person wird wiederholt zufällig einer Aufforderungsbedingung (Encouragement) oder Kontrollbedingung zugeordnet. Die Aufforderung soll ein Verhalten fördern, ohne seine Ausführung vorauszusetzen. Bei einer bewegungsbezogenen Aufforderung lässt sich getrennt prüfen, ob sie Bewegung auslöst und ob die ausgelöste Bewegung anschließend psychisches Erleben verändert [12, 13].

**Abb. 2:**
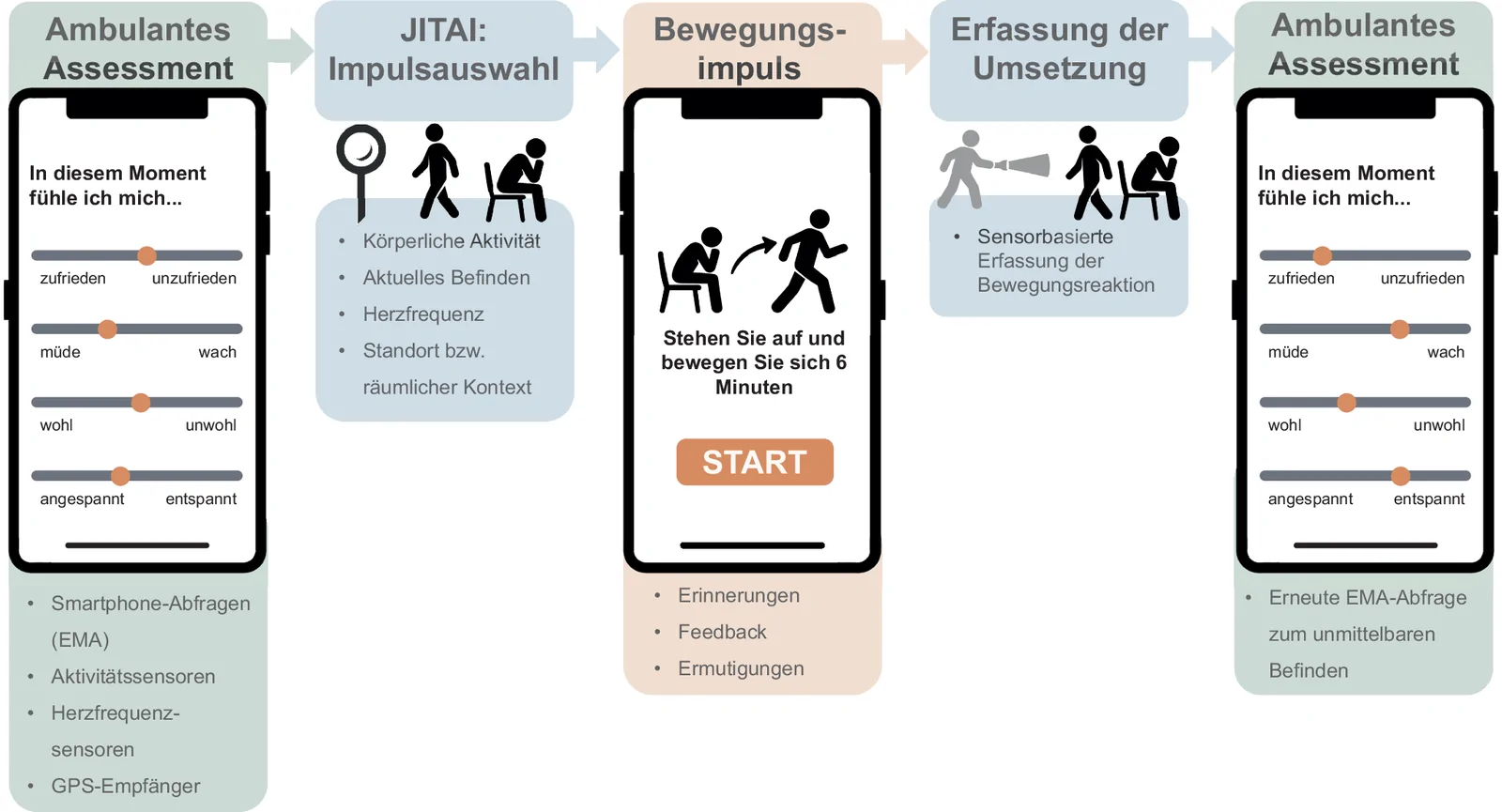
Zusammenspiel von Ambulantem Assessment und einer Just-in-Time Adaptive Intervention (JITAI) zur Bewegungsförderung im Alltag. Smartphonebasierte EMA-Abfragen (Ecological Momentary Assessment) und Sensoren erfassen Befinden, körperliche Aktivität, Herzfrequenz und räumlichen Kontext. Eine JITAI nutzt diese aktuellen Informationen, um einen passenden Bewegungsimpuls auszuwählen. Anschließend werden die Bewegungsreaktion sensorbasiert und das unmittelbare Befinden erneut mittels EMA erfasst. Quelle: eigene Darstellung. GPS: Global Positioning System

Die gewonnenen Erkenntnisse können in Just-in-Time Adaptive Interventions (JITAIs) einfließen. JITAIs nutzen aktuelle Informationen aus Ambulantem Assessment und Sensorik, um Art und Zeitpunkt digitaler Unterstützung an die Situation anzupassen. Für die digitale Bewegungsförderung bedeutet das, einen Bewegungsimpuls möglichst dann anzubieten, wenn er zur Situation passt, und anschließend seine Umsetzung sowie mögliche Folgen zu erfassen. Abb. 2 veranschaulicht diesen Ablauf [7, 11].

### Wearables als technologische Grundlage

Die passive, gerätegestützte Datenerfassung mittels Ambulanten Assessments erfolgt häufig via Wearables. Diese sind am Körper getragene elektronische Geräte, die mithilfe von Sensoren und Software bewegungsbezogene oder physiologische Daten erfassen und verarbeiten. Smartwatches und Fitness-Tracker messen im Alltag beispielsweise Schritte, Bewegungsintensität oder Herzfrequenz [28].

Für die digitale Bewegungsförderung können Wearables Bewegungsverhalten kontinuierlich erfassen und zugleich Interventionen unterstützen. Phasen geringer oder hoher Aktivität können eine EMA-Abfrage auslösen und in JITAIs als Auslöser für Bewegungsimpulse dienen [11, 29]. Funktionen wie Selbstbeobachtung (Self-Monitoring), Rückmeldung, Zielsetzung und Benachrichtigungen sind konkrete Verhaltensänderungstechniken, die körperliche Aktivität fördern können [30].

Systematische Übersichtsarbeiten und Metaanalysen sprechen dafür, dass Interventionen mit solchen Aktivitätstrackern körperliche Aktivität und Fitness steigern können. Je nach untersuchtem Endpunkt wurden im Mittel etwa 1800 zusätzliche Schritte oder rund 40 zusätzliche Gehminuten pro Tag berichtet [31]. Diese Befunde verdeutlichen das Potenzial der Technologie. Werden Sensordaten mit Angaben zum aktuellen Erleben und zur Alltagssituation verknüpft, lassen sich Bewegungsimpulse gezielter an die einzelne Person und den jeweiligen Moment anpassen [7, 11].

Auch in der psychischen Gesundheitsversorgung können Wearables körperliche Aktivität, Schlaf und physiologische Signale im Alltag erfassen. Diese Daten können die klinische Beurteilung und Behandlung depressiver Störungen ergänzen [32]. Für die digitale Bewegungsförderung in diesem Bereich können Daten von Wearables und des Ambulanten Assessments genutzt werden, um Bewegungsverhalten mit dem aktuellen Befinden zu verknüpfen und Bewegungsimpulse an die jeweilige Situation anzupassen [7, 32].

## Von der Beobachtung zur Intervention: Einblicke in die Sucht- und Depressionsforschung

Im Folgenden werden Einblicke in die Beispielprojekte C05 und MASE gegeben. Sie untersuchen aus der Perspektive der Grundlagenforschung, wie alltagsnahe Erhebungen und Sensordaten möglicherweise für situationsangepasste Bewegungsimpulse genutzt werden könnten [13, 14]. Ziel ist es, die Zusammenhänge zwischen Triggern zur Bewegungsförderung, kurzfristiger körperlicher Alltagsaktivität und momentanen psychologischen Korrelaten im Alltag hinsichtlich ihrer zeitlichen Direktionalität und Kausalität zu prüfen.

### Projekt C05.

In der Suchtforschung sind kontextuelle und personenbezogene Faktoren zentral, da Risikosituationen und Rückfallprozesse stark alltags- und situationsabhängig auftreten können. „C05“ ist ein seit 2023 laufendes Teilprojekt des Sonderforschungsbereichs/Transregio 265 (SFB/TRR 265) und wird am Zentralinstitut für Seelische Gesundheit in Mannheim sowie an der Charité – Universitätsmedizin Berlin durchgeführt. In dem Projekt verbindet eine digitale Interventionsplattform die Erfassung von Craving und Einsamkeit im Alltag mit JITAIs [13].

Mittels des WPED werden wiederholt 3 Interventionsbedingungen im Alltag angeboten: eine 6‑minütige bewegungsbezogene Intervention, eine psychotherapeutische Intervention zur Umstrukturierung dysfunktionaler Kognitionen und eine Kontrollbedingung. Bei der Bewegungsintervention wird im ersten Schritt untersucht, ob die digitale Aufforderung tatsächlich Bewegung auslöst. Bewegungssensoren ergänzen dabei die elektronischen Tagebuchabfragen und erfassen, ob die Aufforderung umgesetzt wurde. In einem zweiten Schritt wird geprüft, ob die ausgelöste Bewegung das psychologische Erleben (z. B. Craving) verändert. So veranschaulicht das Projekt, wie Wearables und mobile Datenerhebung situationsangepasste und adaptive Bewegungsförderung im Alltag ermöglichen [12, 13].

**Abb. 3:**
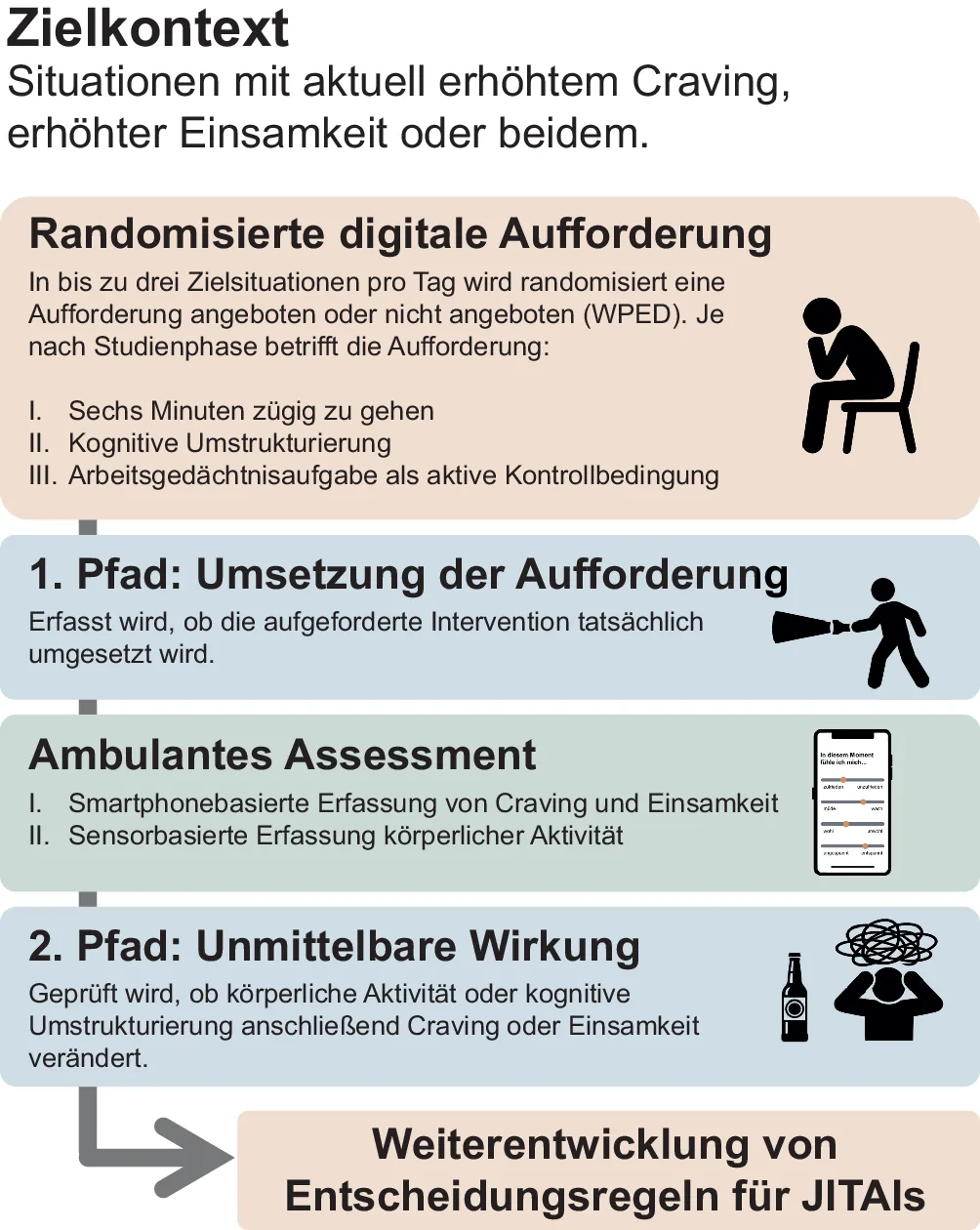
Experimentelle Prüfung digitaler Interventionen im Teilprojekt C05. In Alltagssituationen mit erhöhtem Craving oder erhöhter Einsamkeit wird randomisiert eine digitale Aufforderung angeboten oder nicht angeboten. Das Within-Person-Encouragement Design (WPED) ermöglicht die getrennte Prüfung, ob die Aufforderung umgesetzt wird und ob körperliche Aktivität oder kognitive Umstrukturierung Craving oder Einsamkeit unmittelbar verändert. Die Ergebnisse dienen der Weiterentwicklung von Entscheidungsregeln für Just-in-Time Adaptive Interventions (JITAIs). *Craving* bezeichnet Suchtdruck beziehungsweise das momentane Verlangen nach Alkohol. Quelle: eigene Darstellung auf Grundlage der Studiendesigns von Liu et al. [13] und Haucke et al. [33]

Für die erfolgreiche Anwendung solcher JITAIs sind Entscheidungsregeln erforderlich, die Interventionen etwa bei Craving oder Einsamkeit im passenden Moment auslösen, ohne die Teilnehmerinnen und Teilnehmer durch zu häufige Aufforderungen zu überfordern. Mit einem Simulationsansatz wurden bereits verschiedene Entscheidungsregeln anhand eines bestehenden und eines simulierten Datensatzes unter anderem hinsichtlich ihrer Sensitivität und Spezifität verglichen. Dabei deutete sich das günstigste Verhältnis beider Kennwerte an, wenn bereits bei vorhandenem, aber noch geringem Craving oder geringerer Einsamkeit interveniert wurde. Auch Wochenenden schienen günstige Interventionszeitpunkte zu sein. Wegen großer Unterschiede in den individuellen Trinkmustern dürfte ein Wochenendfokus allein jedoch nicht ausreichen, da beispielsweise Personen mit erhöhtem Alkoholkonsum werktags nach Feierabend dadurch möglicherweise nicht zuverlässig erfasst würden. Die bisherigen Ergebnisse legen nahe, dass auslösende Variablen und Interventionszeitpunkte sorgfältig vorab geprüft und an individuelle Alltagsmuster angepasst werden sollten [33]. Abb. 3 zeigt den Ablauf im Projekt C05.

### MASE-Projekt.

Auch das internationale, im Rahmen des europäischen Forschungsnetzwerks ERA-NET NEURON geförderte MASE-Projekt (Motor Activity – Subjective Energy) untersucht einen vergleichbaren Ansatz. Der deutsche Projektteil wird seit 2024 an der Ruhr-Universität Bochum und am Zentralinstitut für Seelische Gesundheit Mannheim durchgeführt. Ziel ist es, die Wirkung situationsangepasster digitaler Bewegungsimpulse auf psychologisches Erleben bei depressiven Störungen im Alltag zu untersuchen [14].

Depressive Störungen können mit reduzierter körperlicher Aktivität sowie Energie- und Antriebsverlust einhergehen [3, 14]. Ergebnisse aus dem Ambulanten Assessment legen nahe, dass körperliche Alltagsaktivität insbesondere mit einem erhöhten Gefühl von Energie und Antrieb verbunden sein kann [6, 26, 34]. Dabei könnte der subgenuale Anteil des anterioren cingulären Kortex eine wichtige Rolle spielen [35]. Da die Kausalität dieses Zusammenhangs und seine Bedeutung für depressive Störungen bislang unzureichend untersucht sind [34], werden im MASE-Projekt individualisierte bewegungsbezogene Interventionen auf Grundlage kontinuierlicher Alltagsdatenerfassung entwickelt. Dabei sollen mögliche unmittelbare Veränderungen von subjektiver Energie und subjektivem Antrieb durch körperliche Aktivität untersucht und auf Kausalität geprüft werden [14].

Untersucht werden gesunde Personen sowie Personen mit remittierter oder akuter Depression in einer 5‑wöchigen experimentellen Studie auf Grundlage des WPED [12]. Beschleunigungssensoren erfassen die körperliche Aktivität kontinuierlich und dienen zugleich dazu, geeignete Interventionszeitpunkte zu bestimmen.

Registrieren die Sensoren länger als 30 Minuten weniger als 10 Schritte pro Minute, erhalten die Studienteilnehmerinnen und Studienteilnehmer bis zu 4‑mal täglich über das Studiensmartphone randomisiert eine von 3 Aufforderungen: 6 Minuten zügig zu gehen, ein kurzes Arbeitsgedächtnisspiel als aktive Kontrollbedingung zu spielen oder eine weitere Abfrage zum aktuellen Befinden als passive Kontrollbedingung auszufüllen [14]. Anschließend wird untersucht, ob der digitale Bewegungsimpuls tatsächlich zu mehr Bewegung führt.

Im zweiten Schritt wird untersucht, ob die angeregte Alltagsaktivität das Gefühl von Energie und Antrieb unmittelbar verändern kann. Zeit, Ort, sozialer Kontakt und Schlafqualität werden mittels Ambulanten Assessments erfasst, um zu untersuchen, unter welchen Bedingungen die Effekte besonders ausgeprägt sind, und daraus Entscheidungsregeln für künftige Interventionen abzuleiten. Zu Beginn der Studie wird zudem eine Hirnbildgebung durchgeführt, um neurobiologische Merkmale bei der Interventionsgestaltung berücksichtigen zu können.

## Translation in die Regelversorgung: Digitale Gesundheitsanwendungen (DiGA)

DiGA bieten in Deutschland einen möglichen Weg, aus den Erkenntnissen der Grundlagenforschung in vielen weiteren Schritten (u. a. randomisiert-kontrollierte und klinische Studien) entwickelte und geprüfte digitale Angebote zur Bewegungsförderung qualitätsgesichert und zugänglich in die Versorgung zu überführen [36].

DiGA sind keine herkömmlichen Gesundheits-Apps oder Fitnessprogramme, sondern zertifizierte Medizinprodukte, deren Hauptfunktion wesentlich auf digitalen Technologien beruht. Sie werden beispielsweise als Smartphone-App oder browserbasierte Anwendung bereitgestellt und können Patientinnen und Patienten bei der Erkennung, Überwachung oder Behandlung von Krankheiten sowie auf dem Weg zu einer selbstbestimmten gesundheitsförderlichen Lebensführung unterstützen [36].

Vor der Aufnahme in das öffentlich zugängliche DiGA-Verzeichnis prüft das Bundesinstitut für Arzneimittel und Medizinprodukte (BfArM) unter anderem Sicherheit, Funktionstauglichkeit, Qualität, einschließlich der medizinischen Inhalte, Datenschutz und Datensicherheit. Für die dauerhafte Aufnahme ins Verzeichnis müssen positive Versorgungseffekte nachgewiesen sein; zur weiteren Erprobung kann eine DiGA zunächst vorläufig aufgenommen werden. Das Verzeichnis informiert über Indikationen, Funktionen und wissenschaftliche Nachweise. Gelistete DiGA können ärztlich oder psychotherapeutisch verordnet und von den gesetzlichen Krankenkassen erstattet werden [36].

Einzelne DiGA greifen bereits Elemente alltagsnaher Erfassung auf: Digitale Kurzabfragen und Tagebuchfunktionen erfassen Stimmung, Symptome oder Aktivitäten wiederholt im Alltag; Rückmeldungen machen Veränderungen sichtbar, während angepasste Dialoge, Erinnerungen und Übungen Unterstützung außerhalb fester Behandlungstermine bereitstellen [37–39].

Ein Beispiel hierfür ist das evidenzbasierte Programm vorvida, das darauf abzielt, Patientinnen und Patienten mit schädlichem Alkoholkonsum durch kognitiv-verhaltenstherapeutische Ansätze im Alltag zu unterstützen [39, 40]. Die Anwendung hilft ihnen, ihr Trinkverhalten zu reflektieren, persönliche Veränderungsziele festzulegen und mit Alkoholverlangen sowie Risikosituationen umzugehen. Dazu leitet sie die Nutzerinnen und Nutzer an, in Risikosituationen das Verlangen nach Alkohol mithilfe der Achtsamkeitstechnik „Urge Surfing“ wie eine vorübergehende Welle zu beobachten, statt ihm nachzugeben. Auf diese Weise können therapeutische Strategien unmittelbar im Alltag eingeübt werden [39, 40].

Bei depressiven Störungen können Anwendungen wie „deprexis“ und „Selfapy“ therapeutische Behandlungen ergänzen und deren Inhalte in den Alltag übertragen. Beide beruhen auf Elementen der kognitiven Verhaltenstherapie und unterstützen Nutzerinnen und Nutzer durch Psychoedukation, strukturierte Übungen und Selbstbeobachtung [37, 38, 41]. deprexis passt die Dialoge an die jeweiligen Antworten an und bildet Aktivitäten und Symptome im Verlauf ab [37, 42]. Körperliche Aktivität wird im Rahmen der Verhaltensaktivierung sowie gemeinsam mit Entspannung und weiteren Lebensstilthemen aufgegriffen [42, 43]. Selfapy bietet eine Tagebuchfunktion und behandelt in der Lektion „Aktiv werden“ Tagesstruktur, Aktivitäten und den positiven Einfluss von Bewegung auf die Psyche [41].

## Diskussion

Ob digitale Angebote Bewegungsverhalten verändern, hängt nicht allein von der Technologie ab. Entscheidend ist nach aktuellem Wissensstand, welche der zuvor beschriebenen reflektiven und automatischen Prozesse sie berücksichtigen und welche Verhaltensänderungstechniken sie einsetzen. Dazu gehören etwa Selbstbeobachtung, Rückmeldung und Zielsetzung [16, 30]. Bei JITAIs legen Entscheidungsregeln fest, unter welchen Bedingungen und in welcher Form ein Bewegungsimpuls angeboten wird; die Auswahl der Impulse und die Ausgestaltung der Regeln sollten sich an Theorie und empirischer Evidenz orientieren [11]. Eine belastbare Prüfung erfordert valide und reliable Messungen von Bewegungsverhalten und Erleben sowie geeignete Within-Person-Analysen [7]. Experimentelle Alltagsstudien können getrennt untersuchen, ob ein Impuls Bewegung auslöst und ob die angeregte Bewegung anschließend psychisches Erleben verändern kann [12, 13].

Aktuelle Studien zeigen, dass bei Wearables Qualität und Vergleichbarkeit der Messungen zentrale Herausforderungen sind. Eine zusammenfassende Auswertung von 967 Validierungsstudien bewertet deren methodische Qualität insgesamt als gering und verweist auf fehlende standardisierte Validierungs- und Regulierungsprozesse sowie den Bedarf an transparenten Angaben zu den eingesetzten Algorithmen [44].

Bei vorhandenen DiGA unterscheidet sich die Rolle körperlicher Aktivität zwischen den Indikationsbereichen. Während körperliches Training in einzelnen Anwendungen für muskuloskelettale oder urologische Erkrankungen einen zentralen Bestandteil bildet, ist Bewegungsförderung in den hier betrachteten DiGA für schädlichen Alkoholkonsum und depressive Störungen eher ergänzend verankert oder nicht als eigenständiger Baustein beschrieben [36, 43]. Angesichts der Bedeutung körperlicher Aktivität für die psychische Gesundheit deutet dies auf ein bislang nur begrenzt genutztes Potenzial hin [3, 6, 43].

### Limitationen

Der hier vorliegende narrative Überblick beruht nicht auf einer systematischen Literaturrecherche oder einem vollständigen Technology-Scouting. Die Beispiele sind daher nicht ohne Weiteres auf alle Zielgruppen, Technologien und Anwendungsfelder übertragbar. Die Einordnung der DiGA stützt sich auf ausgewählte öffentliche Angaben, in denen Programmbestandteile unterschiedlich detailliert beschrieben werden. Aus einer fehlenden Nennung lässt sich deshalb nicht schließen, dass tatsächlich ein Bestandteil fehlt [43]. Die herangezogenen Wirksamkeitsstudien zu deprexis und Selfapy untersuchen zudem jeweils das Gesamtprogramm und nicht den isolierten Bewegungsanteil [37, 38]. Deshalb lassen sich weder der eigenständige Effekt einzelner Bewegungsanteile noch deren Umfang und Häufigkeit über alle DiGA hinweg verlässlich bestimmen.

Für Ambulantes Assessment und Wearables können geringe Adhärenz sowie fehlende oder verzerrte Daten die Aussagekraft einschränken. Bereits bei der Planung sollten deshalb Erhebungsaufwand, Tragekomfort und Maßnahmen zur Förderung der Teilnahme berücksichtigt werden [7, 45]. Die kontinuierliche Erfassung sensibler Sensor- und EMA-Daten stellt zudem besondere Anforderungen an Datenschutz und Datensicherheit, insbesondere bei klinischen Populationen [7].

Digitale Angebote können die psychotherapeutische Versorgung niedrigschwellig ergänzen. Die therapeutische Allianz, also die Zusammenarbeit zwischen behandelnden Personen und Patientinnen und Patienten, bleibt jedoch ein wichtiger Prädiktor für den Therapieerfolg [46]. Bei rein smartphonebasierten Interventionen werden häufig geringe Adhärenz, Studienabbrüche und eine im Verlauf abnehmende Nutzung berichtet [47]. Auch zusätzliche Bildschirmzeit durch app- oder browserbasierte Angebote sollte gegenüber ihrem therapeutischen Nutzen abgewogen werden. Hohe Bildschirmzeiten sind bei Erwachsenen mit psychischen Belastungen und geringerem Wohlbefinden assoziiert; Art und Zweck der Nutzung sind jedoch bedeutsam, sodass sich diese Befunde nicht ohne Weiteres auf therapeutische digitale Angebote übertragen lassen [48].

### Ausblick

Für die Zukunft der digitalen Bewegungsförderung ergeben sich daraus Forschungs- und Handlungsbedarfe. Die Entscheidungsregeln adaptiver Interventionen müssen weiterentwickelt werden, um einen geeigneten Zeitpunkt für einen Bewegungsimpuls zu bestimmen, ohne die Nutzerinnen und Nutzer durch zu häufige oder unpassende Benachrichtigungen zu überlasten oder Reaktanz auszulösen [11]. Gerade bei DiGA für psychische Erkrankungen sollten bewegungsbezogene Bausteine gezielt entwickelt und ihre spezifischen Effekte gesondert geprüft werden.

Für Wearables sollten standardisierte, gestufte Validierungsverfahren von der technischen Entwicklung und Kalibrierung über Labor- und Alltagsvalidierung bis zum Einsatz in Gesundheitsstudien etabliert werden [49]. Transparente Angaben zur Datenverarbeitung sowie zu den eingesetzten Algorithmen und deren Änderungen sind ebenfalls erforderlich; Open-Science-Initiativen wie „The Wearable Landscape“ können die Bündelung von Validierungsprotokollen und den Austausch zwischen Wissenschaft und Praxis unterstützen [44]. Zudem ist zu untersuchen, für welche Zielgruppen Blended-Care-Konzepte, also die Verknüpfung digitaler Angebote mit persönlicher ärztlicher oder psychotherapeutischer Behandlung, geeignet sind und wie alltagsnahe digitale Unterstützung persönliche ärztliche oder psychotherapeutische Behandlungskontakte sinnvoll ergänzen kann, ohne sie vollständig zu ersetzen [50]. Bei der Weiterentwicklung und Überführung digitaler Angebote in die Versorgung sind zudem ethische Anforderungen, Datenschutz, Datensicherheit und Patientenrechte von Beginn an zu berücksichtigen [18, 36].

## Fazit

Digitale Technologien ermöglichen es, Bewegungsförderung an wechselnde Bedingungen im Alltag einer Person anzupassen. Ambulantes Assessment und Wearables können Bewegungsverhalten, Erleben und Kontext erfassen; das Within-Person-Encouragement Design (WPED) ermöglicht die getrennte Prüfung, ob ein digitaler Impuls Bewegung auslöst und ob die ausgelöste Bewegung psychisches Erleben verändern kann [7, 12].

Digitale Gesundheitsanwendungen (DiGA) bieten einen möglichen Transferweg für digitale Interventionen in die Versorgung, doch in den hier betrachteten DiGA für psychische Erkrankungen ist Bewegungsförderung bislang eher ergänzend verankert oder nicht als eigenständiger Baustein beschrieben [36, 43].

Künftige Ansätze sollten bewegungsbezogene Bestandteile und ihre spezifischen Effekte gezielt prüfen. Voraussetzungen sind valide Messungen und empirisch geprüfte Entscheidungsregeln [7, 11]. Ebenso sollten die Herausforderung der Adhärenz bei smartphonebasierten Interventionen und die Frage, für welche Zielgruppen digitale Unterstützung mit persönlichen Behandlungskontakten verbunden werden sollte, berücksichtigt werden [47, 50].

## Data Availability

Für diesen narrativen Übersichtsbeitrag wurden keine eigenen Datensätze generiert oder analysiert. Die zugrunde liegenden Informationen sind den zitierten Veröffentlichungen zu entnehmen.
